# High fat diet induces gastric production of fibroblast growth factor 23 (FGF23)

**DOI:** 10.1038/s41366-025-01808-3

**Published:** 2025-06-07

**Authors:** Patricia Widmayer, Martina Feger, Leonie Meier, Andreas H. Wagner, Michael Föller

**Affiliations:** 1 Department of Physiology, University of Hohenheim, Stuttgart, Germany; 2 Department of Cardiovascular Physiology, Heidelberg University, Heidelberg, Germany

**Keywords:** Obesity, Calcium and vitamin D

## Abstract

**Background:**

A high fat diet (HFD) leads to lesions of the gastric epithelium and causes a proinflammatory environment. Fibroblast growth factor 23 (FGF23), a bone hormone, regulates renal phosphate and vitamin D metabolism. Under pathophysiological conditions production of FGF23 is stimulated. HFD feeding elevates serum FGF23 levels through inflammation.

**Objective:**

We aimed to investigate whether HFD feeding and obesity is associated with gastric FGF23 production.

**Methods:**

Mice were fed a standard diet or HFD for 12 weeks (long-term) or 1 week (short-term), and the stomachs were then examined. Additionally, corpus specimens from patients with obesity, human umbilical vein endothelial cells (HUVECs), and normal human gastric epithelial cells (GES-1) were studied. FGF23 production was examined by qPCR and Western blotting, mucosal integrity assessed by fluorescence microscopy, and FGF23-expressing cells analyzed by immunohistochemistry.

**Results:**

In mice, HFD feeding up-regulated *Fgf23* expression and FGF23 protein abundance in the proximal glandular stomach. FGF23-positive cells were detected in damaged glandular and interglandular areas representing mucus-like cells, endothelial cells of interconnected blood vessels or stromal endothelial cells and myofibroblasts. FGF23 expression was associated with impaired tissue integrity, immune cell infiltration and lipid deposition, with less pronounced mucosal changes upon short-term HFD feeding. Gastric FGF23 was also detectable in patients with obesity, mainly in endothelial cells of dilated and interconnected vessels. In HUVECs, pro-angiogenic adipokine leptin ramped up *FGF23* transcript levels. In GES-1 cells, proinflammatory cytokine interleukin-1β (IL-1β) tended to enhance *FGF23* expression.

**Conclusions:**

HFD feeding stimulates gastric FGF23 production and, in particular, increases the number of FGF23-expressing endothelial and epithelial cells.

## Introduction

Under physiological conditions, fibroblast growth factor 23 (FGF23) is mainly produced in bone by osteoblasts and osteocytes [1]. Notably, organic phosphate stimulates FGF23 expression and secretion in osteocyte-like cells [2]. FGF23 is also a hormone with paracrine actions [3]. Endocrine functions of FGF23 include the regulation of renal phosphate and vitamin D metabolism [4, 5]: FGF23 reduces membrane abundance of Na^+^-coupled phosphate transporter NaPi-2a encoded by *Slc34a1* and expression of *Cyp27b1*, the key enzyme for the activation of vitamin D [6]. Consequently, FGF23 increases urinary excretion of phosphate and lowers plasma concentration of 1,25(OH)_2_D_3_ (calcitriol), active vitamin D [6].

In contrast to further actions in other organs, these endocrine effects are dependent on renal transmembrane protein αKlotho, serving as a co-receptor for FGF23 [7]. The joint action of FGF23 and αKlotho in the regulation of phosphate and vitamin D metabolism is of high physiological relevance, and FGF23 or αKlotho deficiency results in a phenotype of massively deranged phosphate and vitamin D metabolism with rapid aging and a broad spectrum of aging-associated diseases [8, 9]. Whereas higher αKlotho abundance has been shown to provide health benefits to several organs and confer longevity, higher FGF23 plasma levels are associated with chronic kidney disease (CKD) and further cardiovascular disorders, predicting worse outcomes [5, 10]. Kidney-independent effects of FGF23, including induction of left ventricular hypertrophy, may play a role in this respect [11].

Regulation of FGF23 production is very complex: Under pathophysiological conditions, organs other than bone can produce FGF23, including kidney, heart, liver, and lung [12–15]. Several extracellular (e.g., hormones, cytokines, nutrients) and intracellular factors (e.g., kinases, transcription factors) regulate FGF23 production [5], and FGF23 is subject to posttranslational processing [16].

A high fat diet (HFD) ramps up serum FGF23 levels in mice, an effect at least in part dependent on tumor necrosis factor-α (TNF-α) [17]. The consumption of a diet rich in saturated fatty acids not only promotes the development of obesity but also causes damage to the structure and integrity of various tissues [18] and affects intestinal chemosensing [19]. As the first site of longer contact, excessive intake of saturated fatty acids particularly impacts the stomach [20]. Diet-derived saturated fatty acids are responsible for epithelial impairments: Excess dietary fat causes lipotoxicity to the gastric epithelium, thereby affecting tissue integrity [21–23]. Due to this exposure, ectopic fat predominantly accumulates in surface epithelial cells and disrupts organelle homeostasis [21].

Already in the early course of HFD feeding, the gastric mucosa develops aberrant features characterized by dysregulated gene expression and changes in the cellular composition of the mucosa [21–25]. Morphological lesions primarily manifest in the proximal glandular stomach. They emerge early and worsen with feeding duration. In the course of HFD feeding, multiple distorted abnormal glands (as seen in atrophic gastritis) with metaplastic alterations and structural changes of interglandular spaces occur [21, 23]. Injury-induced metaplastic changes underlie dynamic processes and encompass a range of aberrant gland phenotypes of different metaplastic progression varying from diffuse mucus-producing metaplasia, spasmolytic polypeptide-expressing metaplasia (SPEM) to intestinal metaplasia [26]. Emergence of metaplasia is amplified by a local inflammatory response of immune cell infiltrates involving the production of interleukin-1β (IL-1β) and interleukin-10 (IL-10) [27]. During the development of metaplasia, the expression of key transcription factors, e.g., Sox2 and Cdx2, and of mucins, e.g., Muc5ac and Muc2, which are important for the maintenance of gastric homeostasis, is dysregulated [26]. The emergence of numerous immune cells infiltrating the gastric mucosa is an inflammatory feature of HFD-injured stomachs that has been documented after 3 weeks of HFD feeding [22, 25]. In later stages of HFD feeding, up-regulated proinflammatory cytokine levels of interleukin-6 (IL-6), interleukin-11 (IL-11), and TNF-α contribute to the ongoing gastric inflammatory response [22, 28]. Mucosal damage further impairs gastric vascular homeostasis, which causes the formation of new blood vessels [29]. Angiogenesis is accompanied by increased blood flow, which protects the gastric epithelium by removing injurious metabolites [30].

As highlighted by recent reports, gastric expression of the adipokine leptin significantly augments already within one week of HFD feeding in the stomach [22, 28, 31]. Abundant gastric leptin production is suggested to be necessary for the maintenance of gastric mucosal homeostasis [22]. Importantly, leptin induces FGF23 production in bone [32].

*Fgf23* mRNA expression is low, albeit detectable, in the stomach of healthy mice [33]. Since HFD feeding enhances serum FGF23 levels and causes multiple lesions to the gastric mucosa, we explored whether it affects gastric FGF23 production. We used a murine model of HFD-induced obesity and examined FGF23 expression levels and protein localization in the stomachs. Moreover, we studied FGF23 in gastric specimens from patients with obesity.

## Material and methods

### Animal model and maintenance

The animal experiments in this study were approved by the state of Baden-Württemberg, Germany, and were in accordance with the federal law for welfare of animals.

Male adult mice (B6129PF2/J background, JAX Stock #100903, The Jackson Laboratory, Bar Harbor, ME, USA) were analyzed. No randomization method was used to assign the mice to designated experimental groups. Sample size was adjusted according to recent reports using male mice in HFD feeding models [21, 25]. No animals were excluded from analyses. Investigators were not blinded to group allocation during the experiments. To induce obesity, mice were fed a HFD containing 70% energy from fat (C1090-70, Altromin, Lage, Germany) for 12 weeks (HFD; n = 10). The control group received standard diet (CD; n = 10) with 13% energy from fat. For short-term feeding experiments, mice were fed either CD (CD; n = 5) or HFD (HFD; n = 4) for one week. Throughout the study, mice were housed in groups with free access to chow and water. After the feeding experiment, the animals were sacrificed by cervical dislocation under deep isoflurane anesthesia for tissue sampling.

### Human gastric tissue sample acquisition

Patients with obesity scheduled for laparoscopic sleeve gastrectomy (LSG) in the obesity center Bad Cannstatt (Klinikum Stuttgart) were enrolled and compared to normal-weight subjects. They participated in a clinical trial (German Clinical Trials Register: DRKS00029161). Gastric biopsy specimens from our former study [34] of normal-weight subjects undergoing endoscopy for routine diagnosis served as controls. In total, 6 patients with obesity (3 men, 3 women, body mass index (BMI) 53.7 ± 1.5 ranging from 50.1-58.4, age 45.7 ± 5.3 years ranging from 24-58 years) and 6 normal-weight subjects (3 men, 3 women, BMI 24.5 ± 0.9 ranging from 21.6-27.4, age 41.8 ± 5.6 years ranging from 24-60 years) were analyzed. Exclusion criteria for all subjects were type 2 diabetes, infection with *Helicobacter pylori*, and gastrointestinal disorders.

### Mouse tissue preparation

After removal of the fundus, rinsed stomachs were either used for polymerase chain reaction (PCR) analyses or fixed in 4% buffered paraformaldehyde for immunohistochemistry. After fixation for 2 h at 4 °C, the tissue was cryoprotected in 25% sucrose at 4 °C overnight, embedded in Tissue Freezing Medium (Leica Microsystems, Bensheim, Germany) and quickly frozen in liquid nitrogen. Sections (8 μm) were cut on a CM3000 cryostat (Leica Microsystems) and attached to Superfrost Plus microslides (Menzel Gläser, Braunschweig, Germany). For RNA isolation, narrow strips comprising the uppermost glandular circumference were carefully excised, immediately snap-frozen and stored at −70 °C until use.

### Human tissue preparation

During LSG of patients with obesity, 1$\times$0.5 cm corpus specimens were excised and immediately snap-frozen and stored at −70 °C until use. For immunohistochemistry, samples were fixed in 4% formalin/formaldehyde solution (Roti-Histofix, Carl Roth, Karlsruhe, Germany) for 2 h at 4 °C and processed as described above. As controls, similarly prepared frozen tissue sections of normal-weight subjects were used [34].

### Gastric pH determination

For intragastric pH measurements, a recently described method was adapted [22]. Briefly, stomachs were opened along the greater curvature and crude food was removed. Then, the inner side of the stomach was washed with 400 µl water and the resulting fluid collected. The pH was measured using a pH meter (Mettler Toledo, Albstadt, Germany).

### Histological analysis and immunohistochemistry of the gastric mucosa

Cryosections were stained with periodic acid-Schiff (PAS) using 1% periodic acid (Carl Roth) and Schiff’s reagent (Merck, Darmstadt, Germany) to assess the distribution of neutral gastric mucus.

Prior to immunohistochemical analysis, citrate-antigen-retrieval was carried out (except for IL-1β) by incubating the frozen sections in sodium citrate buffer for 45 min at 4 °C followed by boiling for 1 min at 100 °C. After three washing steps with phosphate-buffered saline (PBS), sections were blocked in PBS containing 10% normal donkey serum and 0.3% Triton X-100 for 1 h at room temperature, followed by an overnight incubation with the primary antibody. For murine sections, the following antibodies at a dilution of 1:400 (except for IL-1β) were used: rat anti-fibroblast growth factor 23 (FGF23) (MAB26291, R&D systems, Minneapolis, MN, USA), mouse anti-hydrogen potassium-ATPase (H^+^/K^+^-ATPase) as parietal cell marker (sc-374094, Santa Cruz Biotechnology, Santa Cruz, CA, USA), mouse anti-platelet endothelial cell adhesion molecule (PECAM/CD31) for endothelial cells (sc-376764, Santa Cruz Biotechnology), rabbit anti-trefoil factor 1 (TFF1) for mucus-producing cells (GTX121461, GeneTex, Irvine, CA, USA), rabbit anti-α-smooth muscle actin (α-SMA) to visualize pericytes and smooth muscle cells (ab5694, Abcam, Cambridge, England), the leukocyte marker rat anti-CD45 to detect immune infiltrates (550539, BD Biosciences, Heidelberg, Germany), and rabbit anti-IL-1β for labeling of infiltrating and epithelial cells (at a dilution of 1:200; P420B, Thermo Fisher Scientific, Darmstadt, Germany).

For human sections, the above-listed rat anti-FGF23 antibody was used (1:100) and for detection of endothelial cells mouse anti-PECAM/CD31 antibody (1:200) (3528 (89C2), Cell signaling, Leiden, Netherlands). Appropriate secondary antibodies conjugated with either Alexa 488 and Cy3 (Dianova, Hamburg, Germany) or to Alexa 555 and Alexa 568 (Thermo Fisher) were diluted 1:500 in blocking solution. After incubation for 2 h at room temperature, sections were rinsed three times in PBS and counterstained with 4,6-diamidino-2-phenylindole (DAPI) (Sigma-Aldrich, Schnelldorf, Germany) to visualize nuclei. Sections were processed without the respective primary antibody to validate immunohistochemical findings. No immunoreactivity was observed in these control experiments. Staining with Nile red (dissolved 1 mg/ml in acetone, Thermo Fisher) was performed using a 1:3000 solution in PBS to visualize lipid deposition.

Immunofluorescence was examined and documented on a Zeiss Axiophot microscope (Carl Zeiss MicroImaging, Jena, Germany). Images were captured using a SensiCam CCD camera (PCO Computer Optics), adjusted for contrast in AxioVision LE Rel. 4.3 (Carl Zeiss MicroImaging) and arranged in PowerPoint (Microsoft; version 16) or Adobe Photoshop (Adobe Systems; version 7.0).

### Morphometric analyses

The proximal mucosal region of the stomach within 3 mm around the lesser curvature was analyzed by acquiring digital microscopic images of sections at 40× magnification. The number of FGF23-positive (FGF23^+^), mucus-like (TFF1^+^) and endothelial cells (PECAM^+^) as well as the quantity of blood vessels were expressed per mm^2^. Relative FGF23^+^/TFF1^+^ areas were determined using ImageJ 1.52p software and given as percentage per mm^2^.

### Luminex assay

Serum concentrations of tumor necrosis factor receptor type 1 (TNFR1) and C-C motif chemokine ligand 2 (CCL2) were quantified using Luminex technology. Therefore, mouse-specific assays from R&D Systems were employed according to the instructions of the manufacturer.

### Qualitative and quantitative polymerase chain reaction of murine samples

Total RNA was isolated from narrow strips of the proximal stomach using peqGOLD TriFast reagent (VWR, Bruchsal, Germany). For cDNA synthesis, 1.2 µg (or 3 µg for *Fgf23* expression analysis) RNA with SuperScript III Reverse Transcriptase (Thermo Fisher Scientific), oligo(dT)_12–18_ and random primers (Promega, Mannheim, Germany) were used on a Biometra TAdvanced thermal cycler (Analytik Jena, Jena, Gemany).

For qualitative expression analysis, RNA from each group was pooled, and 150 ng cDNA used in PCRs with Titanium Taq DNA Polymerase (Takara, Frankfurt, Germany). The cycling profile was: initial incubation at 95 °C for 2 min, then 24 cycles: 30 s at 95 °C, 20 s at 57 °C, and 10 s at 68 °C. Samples processed with intron-spanning primers and without reverse transcriptase (-RT) served as controls for the absence of genomic DNA. PCR products were separated on an agarose gel and stained with ethidium bromide.

Quantitative polymerase chain reactions (qPCRs) were performed using the CFX Connect Real-Time PCR Detection System (Bio-Rad, Feldkirchen, Germany). Each qPCR sample (20 µl per reaction) consisted of a reaction mix containing 0.5 µM of forward and reverse primer, 10 µl GoTaq qPCR Master Mix (Promega), 300 ng cDNA of individual mice, and water. Conditions were: initial incubation at 95 °C for 2 min, then 45 cycles: 95 °C for 15 s, 60 °C (57 °C for *Fgf23*) for 15 s, and 72 °C for 15 s. Melting curve analysis and agarose gel electrophoresis were included to ensure that only a single, specific amplicon was produced. The oligonucleotide primer sequences (5’→3’) were: *Dmp1* sense: GAA CAG TGA GTC ATC AGA AG, *Dmp1* as: AAA GGT ATC ATC TCC ACT GTC (nt 490-685 from GenBank accession number NM_001359013.1), *Fgf23* sense: TCG AAG GTT CCT TTG TAT GGA, *Fgf23* as: AGT GAT GCT TCT GCG ACA AGT (nt 394–524, NM_022657.5), *Il-6* sense: AAG AAA TGA TGG ATG CTA CC, *Il-6* as: GAG TTT CTG TAT CTC TCT GAA G (nt 360-523, NM_001314054.1), *Il-11* sense: TCT CCT AAC CCG ATC CCT CCT G, *Il-11* as: TGC AAA GAT CCC AAT GTC CCA G (nt 284-421, NM_008350), *Rpl8* sense: GTG CCT ACC ACA AGT ACA AGG C, *Rpl8* as: CAG TTT TGG TTC CAC GCA GCC G (nt 600–803, NM_012053.2), *Muc2* sense: AGC CTG GGG AGA TTC ACA AAA ACC, *Muc2* as: ACG GAG ACA GCA GAG CAA GGG A (nt 6691-6907, NM_023566), and *Sox2* sense: TGA CCA GCT CGC AGA CCT ACA TG, *Sox2* as: CGG ACT TGA CCA CAG AGC CCA T (nt 668-778, AB108673), *Tnf-α* sense: GGA TGA GAA GTT CCC AAA TG, *Tnf-α* as: TGA GAA GAT GAT CTG AGT GTG (nt 347-421, NM_013693.3).

### Western blot analysis

Narrow strips of the proximal stomach were lyzed in T-PER tissue protein extraction reagent (Thermo Fisher Scientific) supplemented with complete protease and phosphatase inhibitor cocktail and EDTA (Thermo Fisher Scientific). Proteins (30 µg per lane) were separated on 12% SDS polyacrylamide gels and transferred onto nitrocellulose membranes. For total protein normalization, membranes were stained with Ponceau S solution (AppliChem, Darmstadt, Germany). After destaining, membranes were blocked and subsequently probed with the above-listed rat anti-FGF23 antibody diluted 1:1000 in EveryBlot Blocking Buffer (Bio-Rad) overnight at 4 °C followed by incubation with a HRP-conjugated secondary antibody. Antibody binding was detected with the ECL detection reagent (Bio-Rad) using the ChemiDoc MP Imaging System (Bio-Rad). After normalization to total protein, data were presented as relative fold change of FGF23 expression in CD- versus HFD-fed mice using Image Lab software 6.1 (Bio-Rad).

### Stimulation of cultured endothelial and epithelial cells and expression analysis

For human umbilical vein endothelial cell (HUVEC) cultures, cells were freshly isolated from umbilical cords provided by local hospitals under the approval of the Ethics Committee of the University Hospital Heidelberg (S-383/2013). Cultivation was carried out as previously described [35]. As a gastric epithelial model, the normal human gastric epithelial cell line GES-1 (Cytion, Eppelheim, Germany) was cultured in RPMI 1640 medium supplemented with 10% fetal bovine serum, 100 U/ml penicillin, and 100 μg/ml streptomycin (all from Thermo Fisher Scientific) under standard conditions.

For stimulation of HUVECs, 1.0-1.2 × 10^6^ cells were seeded into 6-well plates, subcultured upon 90*-*95% confluence and incubated with 100 ng/ml recombinant human leptin (Thermo Fisher Scientific) for 48 h. For stimulation of GES-1 cells, 1.5 × 10^5^ cells of passages 17–21 were plated on 6-well plates. After 24 h, GES-1 cells were treated with 100 ng/ml recombinant human IL-1β (Miltenyi Biotec, Bergisch Gladbach, Germany), 50 µM BSA-palmitate saturated fatty acid complex (Cayman Chemicals, Ann Arbor, MI, USA), and 25 ng/ml recombinant human FGF23 (Thermo Fisher Scientific) for 24 h. Treatment with equal amounts of vehicle and BSA control for BSA-fatty acid complexes (Cayman Chemicals) were used as control.

Total RNA was isolated from cells as described above. After DNase treatment (Thermo Fisher Scientific), 2 µg RNA were reverse transcripted (GoScript Reverse Transcription System with random primers (Promega)). Expression of *FGF23* normalized to the internal control encoding either *RPL32* (HUVEC) or *HPRT1* (GES-1) was analyzed by qPCR using the following human primers: *FGF23* sense: GGA TGC TGG CTT TGT GGT GA, *FGF23* as: TGC AGT TCT CCG GGT CGA AAT (nt 364-470, NM_020638.3), *HPRT1* sense: ATA AGC CAG ACT TTG TTG G, *HPRT1* as: ATA GGA CTC CAG ATG TTT CC (nt 606-784, HUMHPRT), and *RPL32* sense: AGG CAT TGA CAA CAG GGT TC, *RPL32* as: GTT GCA CAT CAG CAG CAC TT (nt 194-353, NM_000994.4). Conditions were: initial incubation at 95 °C for 2 min, then 45 cycles: 95 °C for 15 s, 60 °C (58 °C for GES-1) for 15 s, and 72 °C for 15 s.

### Analysis of qPCR data

For analysis of murine samples, relative amounts of transcripts were normalized to *Rpl8* that served as a non-regulated housekeeping gene. For analysis of expression levels in cell culture experiments, the expression of *FGF23* was normalized to the non-regulated housekeeping gene *RPL32* (HUVEC) or *HPRT1* (GES-1). Data from mouse experiments were calculated using the 2^−ΔCt^ method. Relative expression changes of HUVECs and GES-1 cells were calculated using the 2^−ΔΔCt^ method. Data were expressed relative to levels in the vehicle-treated samples (control) and indicated as fold change. For calculations of *FGF23* expression, samples with Cycle threshold (Ct) values ≥ 40, were arbitrarily assigned a Ct value of 40.

### Statistical analysis

Statistical analyses were performed using JASP software (version 0.19.3.0, University of Amsterdam, Amsterdam, Netherlands) or GraphPad Prism 6 (version 6.01; GraphPad Software, Boston, MA, USA). Normality of data distribution was evaluated using Shapiro-Wilk test. Homogeneity of variance was assessed using Levene’s test. Two groups were analyzed with student’s *t*-test, Welch’s *t* test, or Mann-Whitney *U* test. Fold change was analyzed with one-sample Wilcoxon signed rank test. All data are presented as mean ± standard error of the mean (SEM), with *n* indicating the number of individuals per group or independent cell culture experiments. Statistical significance was set at *P* < 0.05.

## Results

### HFD-induced gastric injury causes enhanced FGF23 expression and accumulation of FGF23-expressing cells in gastric structural lesions

**Fig. 1:**
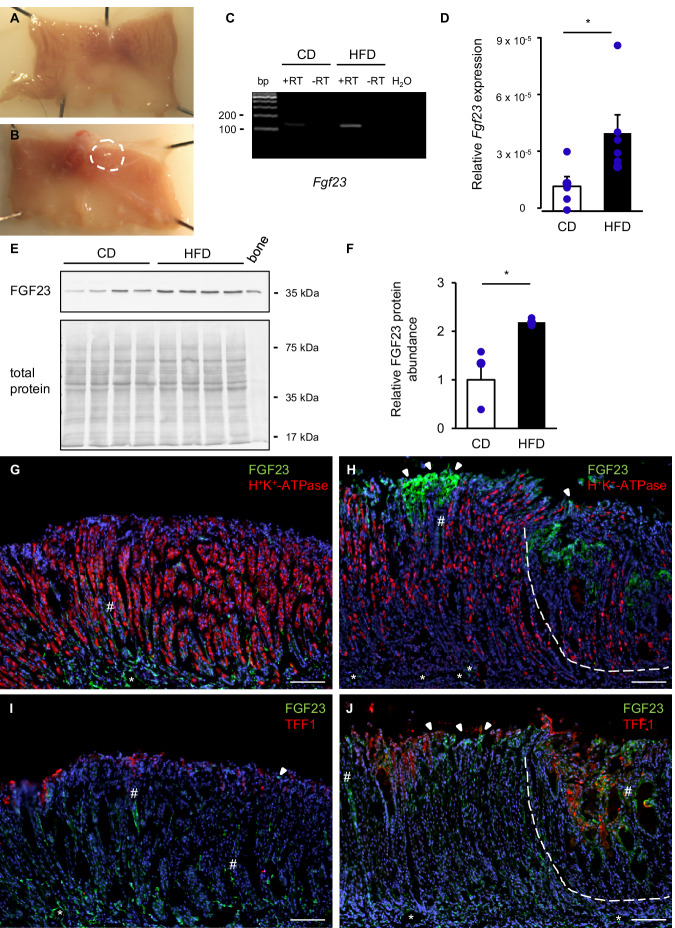
HFD feeding induces gastric lesions, *Fgf23* upregulation and FGF23^+^ cell expansion in the proximal glandular stomach. Original photographs of the opened stomach of a CD-fed mouse with normal gastric mucosa (**A**) and a mouse after 12 weeks on HFD with mucosal abnormalities and poly-like lesions (dashed circle) (**B**). **C** Original gel photograph showing the products of gastric *Fgf23*-specific transcript amplification in the presence (+RT) or absence (-RT) of reverse transcriptase using pooled RNA samples from each group (CD: 44 per group; HFD: 6 per group). **D** Arithmetic means ± SEM of relative gastric *Fgf23* transcript levels normalized to *Rpl8* (CD: n = 5; HFD: n = 6; Mann-Whitney *U* test). **E** Original Western blot showing FGF23 abundance in homogenates from the proximal stomach of CD- and HFD-fed mice using bone as positive control (upper panel) and total protein staining as a loading control (lower panel). Size of marker (in kDa) is shown on the right. **F** Densitometric analysis of FGF23 protein. Data are expressed as fold change of FGF23 protein expression relative to total protein in CD- versus HFD-fed mice and shown as means ± SEM (CD: n = 4; HFD: n = 4; Welch’s *t*-test). Localization of H^+^/K^+^-ATPase (red) and FGF23 (green) in the oxyntic mucosa of the proximal stomach of a CD-fed mouse (**G**) and of a mouse on HFD (**H**). Localization of TFF1 (red) and FGF23 (green) in a CD-fed mouse (**I**) and a mouse on HFD (**J**). A dashed line encircles distended glands, hashmarks point to interglandular spaces, arrowheads to surface epithelium and asterisks to blood vessels (CD: n = 3; HFD: n = 4). **G**–**J** Sections were counterstained with DAPI. Scale bars: 100 μm. *p < 0.05. bp base pairs; CD control diet; FGF23 fibroblast growth factor 23; H^+^/K^+^-ATPase hydrogen potassium-ATPase; HFD high fat diet; *Rpl8* ribosomal protein L8; RT reverse transcriptase; TFF1 trefoil factor 1.

We fed wild-type mice a control diet (CD) or HFD for 12 weeks and observed polyp-like, hyperplastic lesions, as described previously [22, 23]. Briefly, in contrast to CD-fed mice showing no or only local and mild gastric abnormalities (Fig. 1A), stomachs from HFD-fed mice displayed a crooked epithelial surface with pale to glassy, raised gastric lesions. Mucosal lesions notably occurred in the transitional region between the cardia and the corpus of HFD-fed mice (Fig. 1B). Using PCR, HFD-fed mice showed higher *Fgf23* expression compared to control mice (Fig. 1C). We confirmed these results by subsequent qPCR analyses of individual RNA samples, showing a significant increase in *Fgf23* mRNA abundance (p = 0.030) in HFD-fed mice compared to CD-fed mice (Fig. 1D). Western blotting of proximal stomach lysates further confirmed a significant increase in FGF23 protein abundance (p = 0.023) in HFD-fed mice versus CD-fed mice (Fig. 1E, F).

We then determined the localization and distribution of FGF23-expressing cells in the glandular gastric mucosa by performing double-labeling experiments using a mouse-specific FGF23 antibody together with markers for parietal cells (hydrogen potassium-ATPase, H^+^/K^+^-ATPase) and surface mucus cells (trefoil factor 1, TFF1). In the corpus of CD-fed mice a virtually normal mucosal architecture in wide glandular areas was detected as examined by H^+^/K^+^-ATPase (Fig. 1G) and TFF1 (Fig. 1I) staining. In the normal mucosa composition, FGF23-immunopositive cells occurred occasionally, but often in small groups at the mucosal surface (arrowheads), as chained strands of elongated cells along interglandular spaces (hashmarks), or were localized in and around endothelia of blood vessels (asterisks, dotted line) (Fig. 1G, I). Following 12 weeks on HFD, however, the mucosa of cardia and corpus showed severe histopathological changes characteristic of atrophic gastritis and gastric structural lesions. We observed abnormal mucosal structures with disorganized glands and loss of parietal cells (Fig. 1H) paralleled by a trend towards a higher gastric pH in HFD-fed mice compared to CD-fed mice (CD: 2.88 ± 0.24 (n = 4); HFD: 4.76 ± 0.39 (n = 3); p = 0.057). Glands were distended and/or cystic reminiscent of diffuse mucus-producing metaplasia. Overlying foveolae were hyperplastic and populated by numerous cuboidal and columnar TFF1^+^ surface mucus cells (Fig. 1J). An accumulation of FGF23^+^ cells was detected including distended glands (Fig. 1H, J), interglandular spaces and top parts of foveolae (Fig. 1H, J, arrowheads). FGF23 immunoreactivity was apparent in endothelial cells of abundant blood vessels in both groups, with a higher abundance in HFD-fed mice (Fig. 1G-J) suggesting that distinct mucus-secreting, stromal and endothelial cells may be the source of stomach FGF23 expression upon HFD feeding.

### Patchy and extended mucosal lesions harbor FGF23-expressing mucus-like cells

**Fig. 2:**
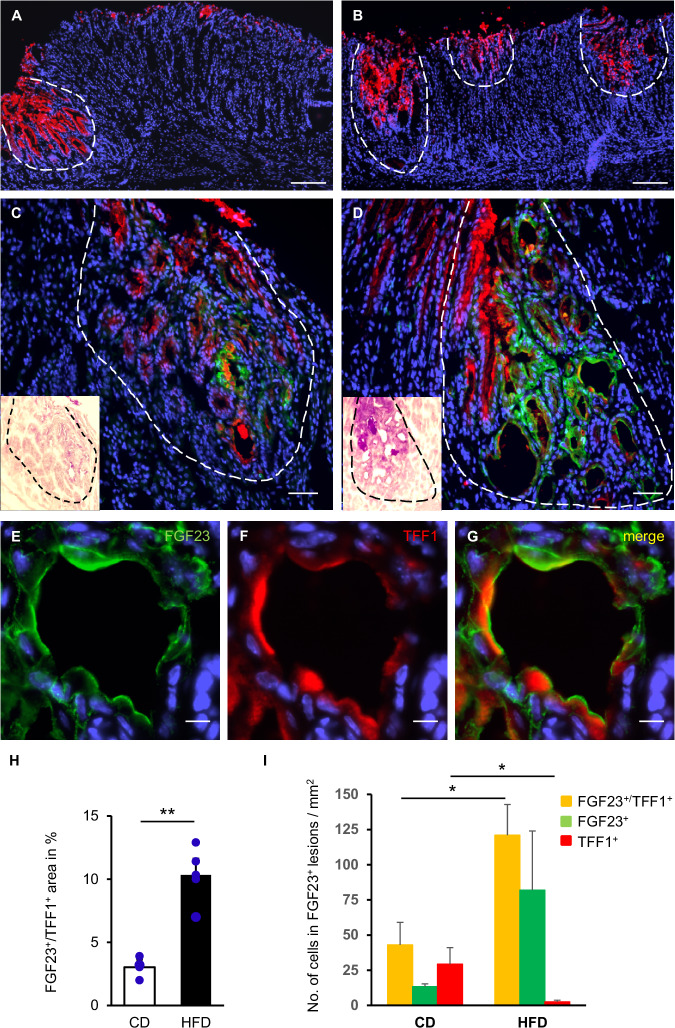
HFD-induced appearance of FGF23-expressing mucus-like cells in gastric lesions. Epithelial lesions visualized by TFF1 staining in CD-fed mice (**A**) and mice on HFD (**B**). Coimmunolabeling of FGF23 (green) and TFF1 (red) in patchy disorganized glands in the stomach of a CD-fed mouse (**C**) and of a mouse on HFD (**D**). Insets: PAS staining verifies the presence of mucin in gastric lesions. Extensive purple color indicates pronounced mucus production, most prominently in elongated foveolae and some dilated glands in the midportion of the mucosa. **E**-**G** Higher magnification of the stomach from a mouse on HFD. (CD: n = 3; HFD: n = 4). **H** Arithmetic means ± SEM of FGF23-/TFF1-positive areas expressed as proportion of total mucosal thickness (CD: n = 3; HFD: n = 4; student’s *t*-test). **I** Arithmetic means ± SEM of the number of cells positive for the indicated markers counted in mucosal lesions (CD: n = 3; HFD: n = 4; cells positive for FGF23/TFF1 and TFF1: student’s *t*-test, cells positive for FGF23: Welch’s *t*-test). A dashed line encircles epithelial lesions. Sections were counterstained with DAPI. Scale bars: 100 µm (**A**, **B**), 50 μm (**C**, **D**), 10 μm (**E**–**G**). *p < 0.05; **p <0.01. CD control diet, FGF23 fibroblast growth factor 23, HFD high fat diet, PAS periodic acid-Schiff, TFF1, trefoil factor 1.

We next examined the abundance of epithelial lesions and the cellular source of FGF23 in diffuse mucus-producing metaplastic areas using an antibody against TFF1, a trefoil factor associated with mucus [36]. Along the lesser curvature of CD-fed mice, TFF1^+^ epithelial patches were evident (Fig. 2A). In contrast, numerous lesions were detectable around the cardiac-corporal transition of stomachs from HFD-fed mice (Fig. 2B). In CD-fed mice, FGF23 immunoreactivity was sparsely observed and overlapped with TFF1 (Fig. 2C). In HFD-fed mice, FGF23^+^/TFF1^+^ lesions were more prominent and dilated glands exhibited huge amounts of mucus (Fig. 2D, inset). In contrast, less mucus content was evident in control mice (Fig. 2C, inset). As depicted in Fig. 2E-G, the overlap of FGF23 and TFF1 was pronounced in dilated glands of HFD-fed mice. We determined the percentage of FGF23-/TFF1-positive areas (Fig. 2H) and the coexpression of FGF23 and TFF1 (Fig. 2I) and revealed that mucosal lesions around the lesser curvature covered significantly more area in HFD-fed compared to CD-fed mice (p = 0.005) (Fig. 2H). In HFD-fed mice, FGF23-stained cells were frequent and detectable in significantly more TFF1-positve mucus-producing cells than in CD-fed mice (p = 0.044) (Fig. 2I). Interestingly, among FGF23^+^ cells a high proportion was negative for TFF1 in mice on HFD, while more cells in control mice expressed only TFF1 (p = 0.044). Hence, HFD induces metaplastic changes with FGF23-positive mucin-rich cells.

### Higher number of FGF23-expressing endothelial cells in blood vessels following HFD

**Fig. 3:**
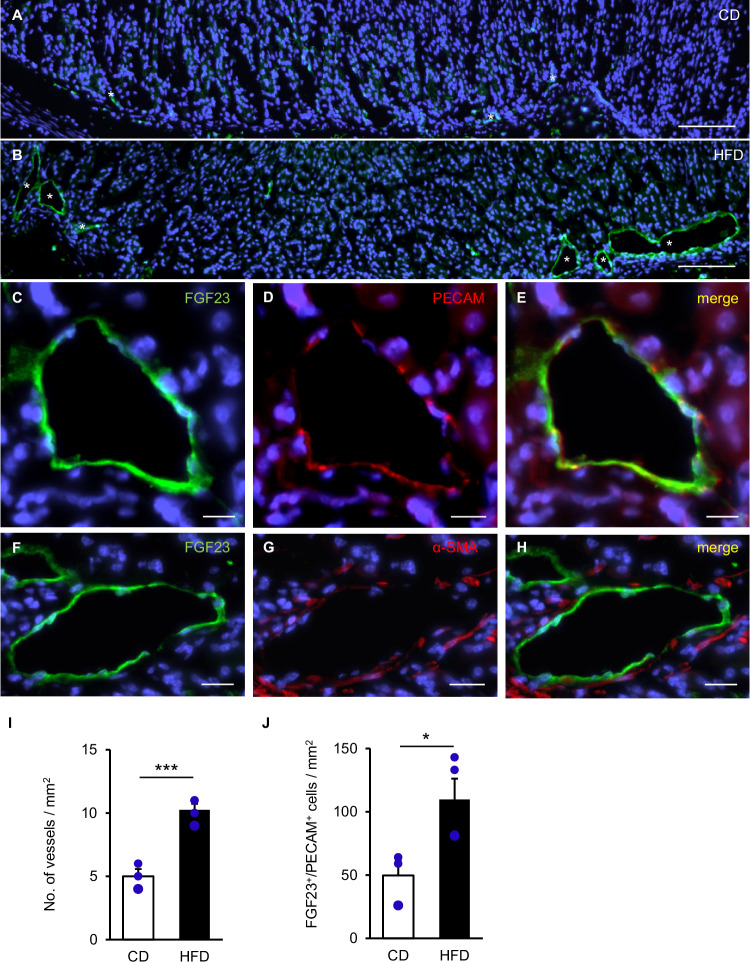
HFD-induced blood vessel formation associated with numerous FGF23-expressing endothelial cells. Distribution of blood vessels in the proximal glandular stomach from a CD-fed mouse (**A**) and a mouse on HFD (**B**). Staining for FGF23 (**C,** **F**), PECAM (**D**), and α-SMA (**G**) and the merge of FGF23 and PECAM (**E**) or FGF23 and α-SMA (**H**) staining in the proximal glandular stomach from a mouse on HFD. (**A**–**H**: CD: n = 3; HFD: n = 4). Arithmetic means ± SEM of the density of FGF23-/PECAM-positive blood vessels (CD: n = 3; HFD: n = 4; student’s *t*-test) (**I**) and of the number of endothelial cells costained for FGF23 and PECAM (CD: n = 3; HFD: n = 4; Welch’s *t*-test) (**J**). Asterisks point to blood vessels. Sections were counterstained with DAPI. Scale bars: 100 μm (**A**, **B**), 10 μm (**C**–**H**). *p < 0.05; ***p < 0.001. α-SMA α-smooth muscle actin, CD control diet, FGF23 fibroblast growth factor 23, HFD high fat diet, PECAM platelet endothelial cell adhesion molecule.

Comparison of immunostainings for FGF23 demonstrated a higher abundance of blood vessels in mice on HFD than on CD (Fig. 3A, B). The overlap of platelet endothelial cell adhesion molecule (PECAM) and FGF23 showed that FGF23-expressing endothelial cells form the inner lining of the blood vessels (Fig. 3C-E) surrounded by few FGF23^+^ α-smooth muscle actin (α-SMA)-immunoreactive pericytes and/or smooth muscle cells (Fig. 3F-H). The higher abundance of blood vessels (p < 0.001) (Fig. 3I) was paralleled by doubling of FGF23^+^/PECAM^+^ cell counts (p = 0.032) (Fig. 3J).

### Distribution of FGF23-expressing cells in the stroma

**Fig. 4:**
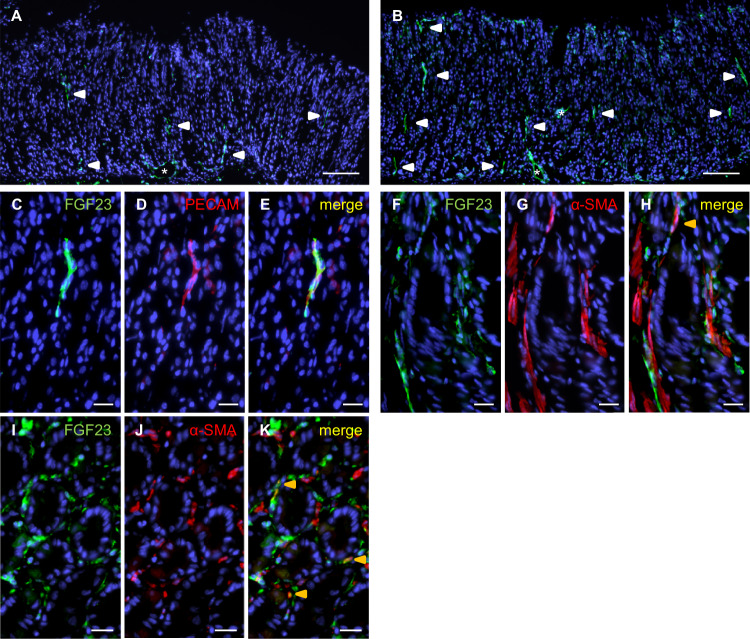
HFD-feeding enhances FGF23 immunoreactivity within periglandular cells of the gastric stroma. Staining for FGF23 in the gastric interglandular spaces of a CD-fed mouse (**A**) and a mouse on HFD (**B**). Immunostaining for FGF23 (**C,** **F,** **I**), PECAM (**D**), α-SMA (**G,** **J**), and merge of FGF23 and PECAM (**E**) or FGF23 and α-SMA (**H,** **K**) staining in interglandular spaces of the corpus (**C**-**H**) and around cardia glands (**I**–**K**) from a mouse on HFD. (CD: n = 3; HFD: n = 4). Asterisks point to blood vessels, white arrowheads to stromal endothelial cells, and yellow arrowheads to myofibroblasts. Sections were counterstained with DAPI. Scale bars: 100 μm (**A**, **B**), 20 μm (**C**–**K**). α-SMA α-smooth muscle actin, CD control diet, FGF23 fibroblast growth factor 23, HFD high fat diet, PECAM platelet endothelial cell adhesion molecule.

Analysis of interglandular spaces revealed that FGF23^+^ cells were sparsely distributed within the corpus mucosa of control mice (Fig. 4A). In contrast, HFD increased the occurrence of chained strands of elongated FGF23^+^ cells in the interstitium of the corpus mucosa (Fig. 4B) with colocalization of FGF23 and PECAM mostly present in 2–4 parallel strands (Fig. 4C-E) representing endothelial cells of microvessels running along glands.

Interestingly, in HFD-fed mice single chains of FGF23^+^ cells were observed along with α-SMA^+^ cells, and few occasionally overlapped (Fig. 4F-H). A comparable relationship for FGF23^+^ and α-SMA^+^ cells was found around distinct cardiac glands of HFD-fed mice: Closely associated FGF23^+^ and α-SMA^+^ cells, possibly myofibroblasts, enwrapped individual glands or in part even displayed immunoreactivity for both (Fig. 4I-K).

### Signs of FGF23^+^ damaged mucosal areas

**Fig. 5:**
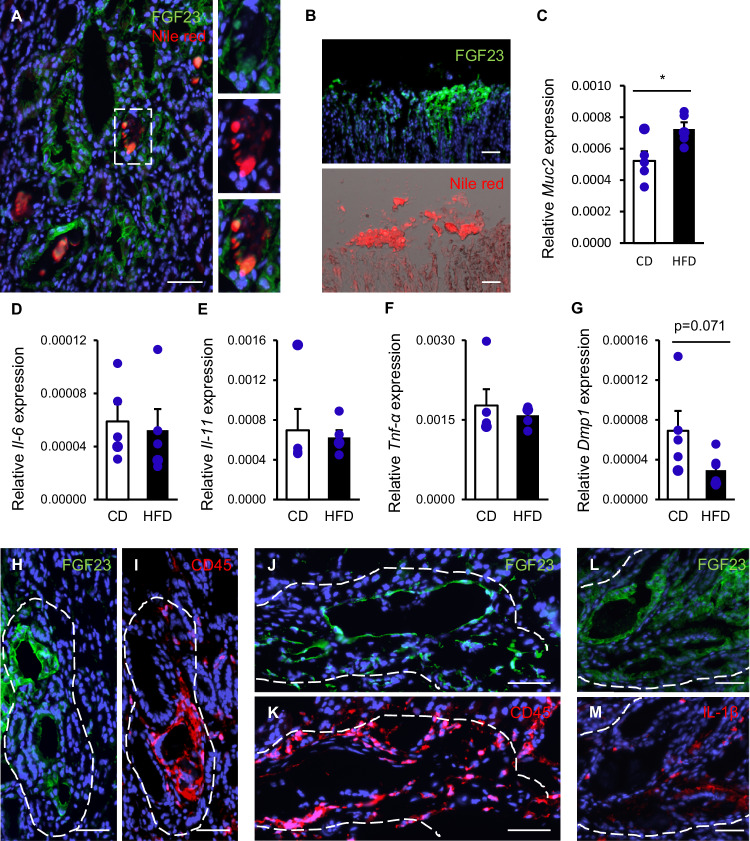
Signs of lipotoxicity, impaired mucosal integrity and local inflammation in FGF23^+^ mucosal areas. **A** Lipid deposits stained by Nile red in metaplastic glands. The right panels indicate the framed area with droplets in FGF23^+^ cells at higher magnification. **B** The upper panel shows FGF23^+^ surface mucus and desquamated cells, displaying strong Nile red staining (lower panel) in the contiguous sections. (CD: n = 3; HFD: n = 4). Arithmetic means ± SEM of gastric *Muc2* (**C**), *Il-6* (**D**), *Il-11* (**E**), *Tnf-α* (**F**) and *Dmp1* (**G**) transcript abundance relative to *Rpl8* in CD- and HFD-fed mice (n = 5-6 per group; **C**, **D**, **G** student’s *t*-test, **E**, **F** Mann-Whitney *U* test). FGF23^+^ metaplastic gland area (**H**) surrounded by CD45^+^ immune cells (**I**) of consecutive sections. Contiguous sections showing dilated FGF23^+^ interconnected blood vessels (**J**) enwrapped by CD45^+^ infiltrates (**K**). FGF23^+^ disorganized glands (**L**) contained IL-1β^+^ inflammatory and epithelial cells (**M**). (CD: n = 3; HFD: n = 4). Sections were counterstained with DAPI. Scale bars (**A**, **B**, **H**–**M**): 50 μm. *p < 0.05. CD control diet, CD45 cluster of differentiation 45, *Dmp1* dentin matrix protein 1*,* FGF23 fibroblast growth factor 23, HFD high fat diet, IL-1β interleukin 1 β, *Il-6* interleukin 6, *Il-11* interleukin 11, *Muc2* mucin 2, *Rpl8* ribosomal protein L8, *Tnf-α* tumor necrosis factor-α.

We further studied lipotoxicity, impaired mucosal integrity, regulators of FGF23 expression and inflammation. Using Nile red, we observed lipid deposits in the lumen and FGF23^+^ cells of some metaplastic glands (Fig. 5A). A patchy Nile red labeling was also seen on the surface of FGF23^+^ gastric epithelial cells and in desquamated overlays (Fig. 5B). In line with the development of a diffuse mucus-producing metaplasia, the expression of *Muc2*, a mucin associated with metaplastic changes, was up-regulated in HFD-fed mice (p = 0.027) (Fig. 5C). Inflammatory cytokines *Il-6*, *Il-11* and *Tnf-α* were not significantly different (Fig. 5D-F), while FGF23 suppressor *Dmp1* was down-regulated by trend (p = 0.071) (Fig. 5G). In particular, FGF23^+^ metaplastic gland areas (Fig. 5H) and FGF23^+^ dilated vessels (Fig. 5J) were surrounded by CD45^+^ inflammatory cells (Fig. 5I, K). Overall, the abundance of infiltrating CD45^+^ cells was more pronounced in HFD-fed than in CD-fed mice (Fig. S1), indicating that HFD feeding impaired mucosal integrity with accumulation of FGF23^+^ cells, immune cells and lipid deposits. Proinflammatory IL-1β implicated in promoting epithelial remodeling turned out to be present in a subset of infiltrating immune cells and some epithelial cells (Fig. 5L, M).

### Occurrence of FGF23-expressing cells and immune cells in mild mucosal lesions after one week on HFD

**Fig. 6:**
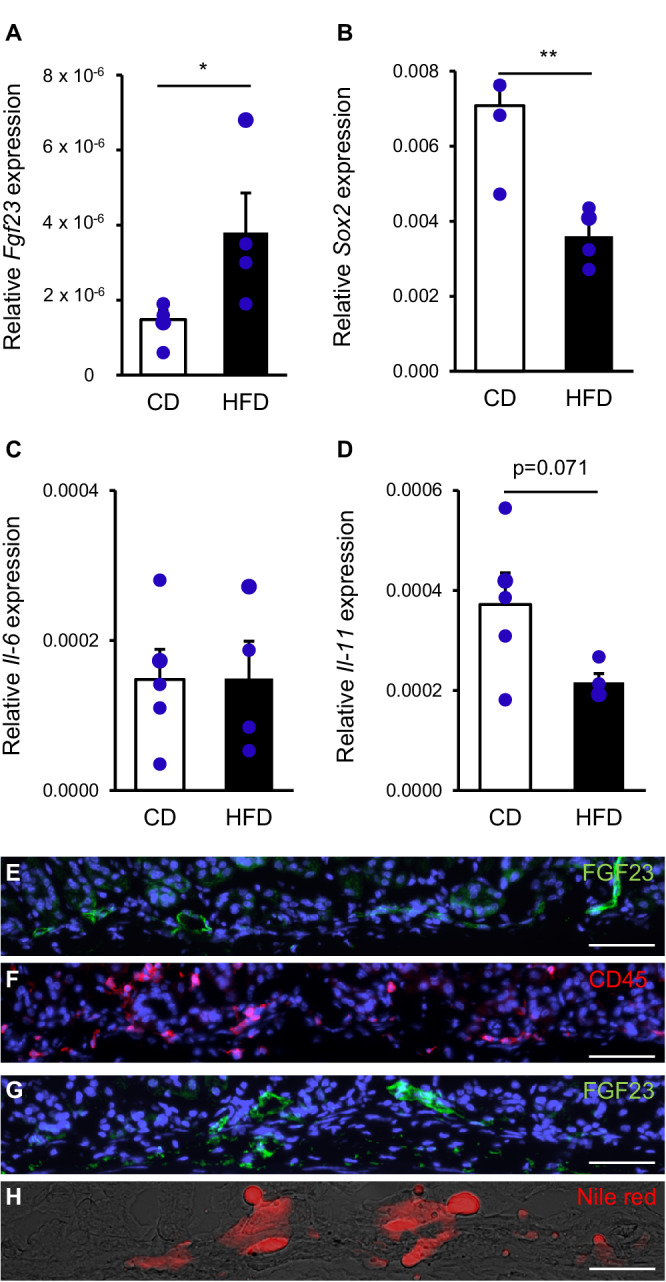
One week on HFD induces upregulation of FGF23 and mild gastric lesions harboring FGF23^+^ cells, CD45^+^ immune cells and lipid accumulation. Arithmetic means ± SEM of relative gastric transcript levels of *Fgf23* (**A**), *Sox2* (**B**), *Il-6* (**C**), and *Il-11* (**D**) normalized to *Rpl8* after one week on HFD compared to CD-fed mice (CD: n = 5; HFD: n = 4; student’s *t*-test). Contiguous sections showing the formation of narrow-lumened FGF23^+^ interconnected blood vessels (**E**) surrounded by CD45^+^ cells (**F**). FGF23^+^ blood vessel (**G**) containing Nile red-stained lipid deposits (**H**) of contiguous sections. (CD: n = 5; HFD: n = 4). Sections were counterstained with DAPI. Scale bars (**E**–**H**): 50 μm. *p < 0.05; **p < 0.01. bp base pairs, CD control diet, CD45 cluster of differentiation 45, *Fgf23* fibroblast growth factor 23, HFD high fat diet, *Il-6* interleukin 6, *Il-11* interleukin 11, *Rpl8* ribosomal protein L8, *Sox2* SRY-box transcription factor 2.

Already within one week of HFD feeding, *Fgf23* expression was significantly enhanced (p = 0.047) (Fig. 6A), and expression of *Sox2*, a transcription factor important for the maintenance of gastric homeostasis, down-regulated (p = 0.003) (Fig. 6B). *Muc2* transcription was not significantly affected (data not shown). Short-term HFD feeding caused superficial injury with foveolar hyperplasia, whereas more profound mucosal injury was less substantial (Fig. S2A, B). Mucosal changes were associated with FGF23 positivity (Fig. S2A). Systemic and local inflammation was not significantly affected (Fig. 6C, D; Fig. S2E, F) as was not CD45 abundance (Fig. S2C,D). CD45^+^ cells were only around focal lesions with multiple FGF23^+^ cells (data not shown). HFD-induced mucosal damage affected the vasculature resulting in interconnected blood vessels (Fig. 6E). FGF23^+^ endothelial cells were juxtapositioned to CD45^+^ cells, enwrapping this capillary network (Fig. 6F). Whereas in both groups no apparent Nile red staining of foveolar cells was detectable (data not shown), in some blood vessels Nile red-stained lipid deposits were seen (Fig. 6G, H). Taken together, these results indicate an inflammatory environment around focal FGF23^+^ injured areas already within one week of HFD.

### FGF23 expression in endothelial and epithelial cells in the human stomach

**Fig. 7:**
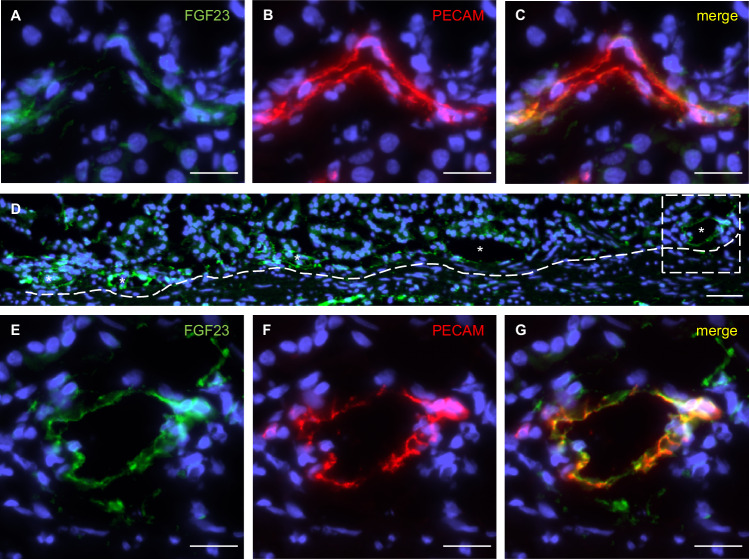
Endothelial cells in the human stomach express FGF23. **A**–**C** Blood vessels in the stomach of a normal-weight subject showing endothelial cells faintly immunoreactive for FGF23 (**A**) but strongly for PECAM (**B**). **D** Gastric blood vessels with interconnections at the base of gastric glands in patients with obesity. **E**–**G** Gastric endothelial cells of a patient with obesity display strong immunoreactivity for FGF23 (**E**) and PECAM (**F**). (Normal-weight: n = 6; patients with obesity: n = 6). A dotted line marks interconnected blood vessels. Sections were counterstained with DAPI. Scale bars: 20 μm (**A**-**C**, **E**-**G**), 50 μm (**D**). FGF23 fibroblast growth factor 23, PECAM platelet endothelial cell adhesion molecule.

Next, we investigated corpus specimens from patients with obesity in comparison to normal-weight subjects. FGF23 was most abundant in endothelial cells located at the base and along gastric glands (Fig. 7). FGF23 was also present in distinct periglandular stromal cells, likely myofibroblasts, whereas rare foveolar cells displayed a faint staining (data not shown). While in normal-weight subjects, endothelial cells of single blood vessels were weakly FGF23-immunoreactive (Fig. 7A-C), FGF23 staining of interconnected blood vessels was more pronounced in patients with obesity indicating a higher vascularization (Fig. 7D-G).

Our study thus far unraveled endothelial cells at least in part accounting for enhanced gastric FGF23 production upon HFD feeding. Since HFD feeding of mice increases gastric leptin, a well-established stimulator of FGF23 production [32], we conducted further experiments in HUVECs to test whether leptin may be a regulator of *FGF23* expression in endothelial cells. HUVECs were incubated with or without 100 ng/ml leptin and *FGF23* transcripts measured after 48 h by qPCR. Figure S3A shows that leptin significantly up-regulated *FGF23* gene expression within 48 h (p = 0.039). Another source of gastric FGF23 in HFD-fed mice were epithelial mucus-like cells. Hence, we performed further experiments in human gastric epithelial cells GES-1. Neither a 24 h incubation with BSA-palmitate nor with recombinant FGF23 nor with both significantly affected *FGF23* gene expression of the cells (Fig. S3B). However, since IL-1β has already been shown to be an inducer of FGF23 [37] and we found it to be strongly expressed in the stomach from HFD-fed mice (Fig. 5M), we exposed GES-1 cells to IL-1β for 24 h and again determined *FGF23* transcripts. As illustrated in Fig. S3C, IL-1β tended to up-regulate *FGF23* expression, a difference almost reaching significance (p = 0.063).

## Discussion

Our study revealed that a diet rich in saturated fat ramps up FGF23 mRNA as well as protein abundance in the proximal glandular stomach. FGF23 upregulation was more pronounced in response to a 12-week HFD intervention compared to one week of HFD feeding. Gastric FGF23 production mainly occurred in damaged gastric epithelial areas exhibiting abundant gastric cell types expressing FGF23 and vicinal immune cells. Within HFD-induced aberrant glandular and interglandular regions, FGF23-immunoreactive cells were characterized as either epithelial mucus-like cells or endothelial cells of blood vessels and stromal endothelial cells as well as myofibroblasts.

Under physiological conditions, FGF23 is mainly expressed in bone cells (osteoblasts/osteocytes) [1]. Compared to bone, FGF23 expression in other organs is low. However, under pathophysiological conditions, FGF23 production in other organs may be ramped up, including kidney in polycystic kidney disease [38], heart in cardiac pathologies [13, 39], or bone marrow in iron deficiency anemia [40]. Whereas an earlier report found very low but detectable gastric FGF23 mRNA expression in the absence of disease [33], our study was the first one demonstrating enhanced gastric FGF23 production under pathophysiological conditions, to the best of our knowledge.

A HFD causes multiple lesions to the gastric mucosa. HFD feeding and obesity are linked to a proinflammatory milieu, and HFD-induced changes of the gastric mucosa are associated with enhanced inflammation [22, 25, 28]. Importantly, inflammation is a major trigger of FGF23 production [41]. Moreover, HFD feeding enhances FGF23 production through inflammation [17]. Within one week of HFD feeding, damaged gastric tissue was found to be paralleled by FGF23 and immune cells, suggesting local inflammatory responses although proinflammatory cytokine expression was not significantly altered. It is therefore intriguing to speculate that local inflammation in the stomach induced by HFD feeding accounted, at least in part, for the upregulation of gastric FGF23 production. In line with this, we found IL-1β expression in the FGF23^+^ damaged mucosa. IL-1β is an inducer of FGF23 in bone cells [37], and our experiments in gastric epithelial GES-1 cells suggested that it may, at least in part, stimulate gastric FGF23 production, too. Furthermore, DMP1 is an important suppressor of FGF23 in bone [42], and we uncovered a trend towards down-regulated *Dmp1* transcript levels in the stomach, a result that may further explain enhanced FGF23 expression following HFD. Finally, our experiment with HUVECs identified leptin as potential trigger for the upregulation of FGF23 in endothelial cells. Since leptin is elevated in the stomach of mice in response to a HFD [22] and in patients with obesity [43], it appears to be possible that enhanced gastric leptin might contribute to gastric FGF23 production. The adverse effects of leptin on the vasculature at higher doses [44] raise the possibility that leptin induces FGF23 upregulation in an already damaged endothelium, further impairing vascular functionality, e.g., by promoting the development of vascular lesions. These results suggest that HFD feeding induces different changes in the stomach that altogether enhance FGF23 producition in different cell types.

Short- and long-term HFD feeding led to many gastric blood vessels with FGF23 positivity. Several interconnected blood vessels were also evident in some stomach samples of patients with obesity. These observations might suggest the requirement of microvasculature expansion due to adaptations of endothelial linings. An important adjustment may concern the permeability of blood vessels, which is increased in response to an inflammatory environment [45].

Our study did not uncover the functional role of gastric FGF23. Higher densities of FGF23-expressing cells in HFD-fed mice and in patients with obesity could point to a local demand for FGF23 production in the changing gastric epithelium. Through paracrine actions, FGF23 may be involved in regulating HFD-induced and obesity-related gastric pathophysiological processes. In line with this, FGF23, locally produced in the failing heart, induces left ventricular hypertrophy [11, 46]. In the liver, local FGF23 induces inflammation [47] and fibrosis [48]. Such injury-induced physiological sequences coordinate tissue repair and involve a network of specialized cells occurring at sites of tissue injury. In the stomach, these are proliferating foveolar mucus cells that migrate to injured sites, reprogrammed glandular cells that convert into mucin-secreting cells and transiently appearing activated fibroblasts (myofibroblasts) remodeling extracellular matrix [44, 49]. Gastric FGF23 may play a role in this process.

Notably, stroma components, such as fibroblasts and myofibroblasts are important for the coordination of the balance between cell proliferation and directional differentiation of gastric gland cells [50]. The finding that myofibroblasts also produce FGF23 in the stomach of mice on HFD could suggest that FGF23 might act as a biochemical signal for cell-cell communication between the gastric stromal and glandular compartment. However, compared to other cells in the stomach, myofibroblasts are likely of minor relevance for FGF23 production.

It is a clear limitation that the number of patients included in our study was small. Moreover, it has to be kept in mind that comorbidities such as T2DM are common in subjects with obesity and that these may also impact FGF23 production. As a matter of fact, insulin has already been found to negatively regulate FGF23 [51], and patients with T2DM are characterized by enhanced FGF23 serum levels [52]. It is intriguing to speculate that gastric FGF23 may contribute to the elevated FGF23 levels, at least in patients with severe obesity. Our study did not reveal whether enhanced gastric FGF23 production in obesity drives pathophysiological processes or which role it has. Since anti-FGF23 therapy is, however, already available for patients [53], further studies are warranted to elucidate whether such therapy is beneficial in obesity.

Also the animal study was small in sample size which is another clear limitation of this study possibly impacting statistical power and generalizability. Our study only included male mice to avoid the impact of female sexual hormones on metabolism. In contrast, our human study also included women, yielding no gross sex difference. However, since subtle differences can not be excluded, further studies are needed.

A recent study revealed an association of Vitamin D status with serum lipids [54]. Since FGF23 is closely related to vitamin D metabolism, further studies on a possible association of FGF23 with vitamin D are additionally desirable.

In summary, we show that HFD-fed mice and patients with obesity exhibit FGF23-expressing cells in altered gastric areas. In particular, endothelial cells of blood vessels along with epithelial mucus-like cells may account for enhanced gastric FGF23 production.

## Supplementary information

Supplemental Material

## Data Availability

The authors confirm that the data supporting the findings of this study are available within the article and its supplementary material.
