# Supplementary material for: High fat diet induces gastric production of fibroblast growth factor 23 (FGF23)
Source: Int J Obes (Lond). 2025 Jun 7;49(9):1733–44. doi: 10.1038/s41366-025-01808-3 (PMC12463679; doi:10.1038/s41366-025-01808-3)
Supplement: Supplementary file 1 — Supplemental Material [file 41366_2025_1808_MOESM1_ESM.pptx]

## Slide 1
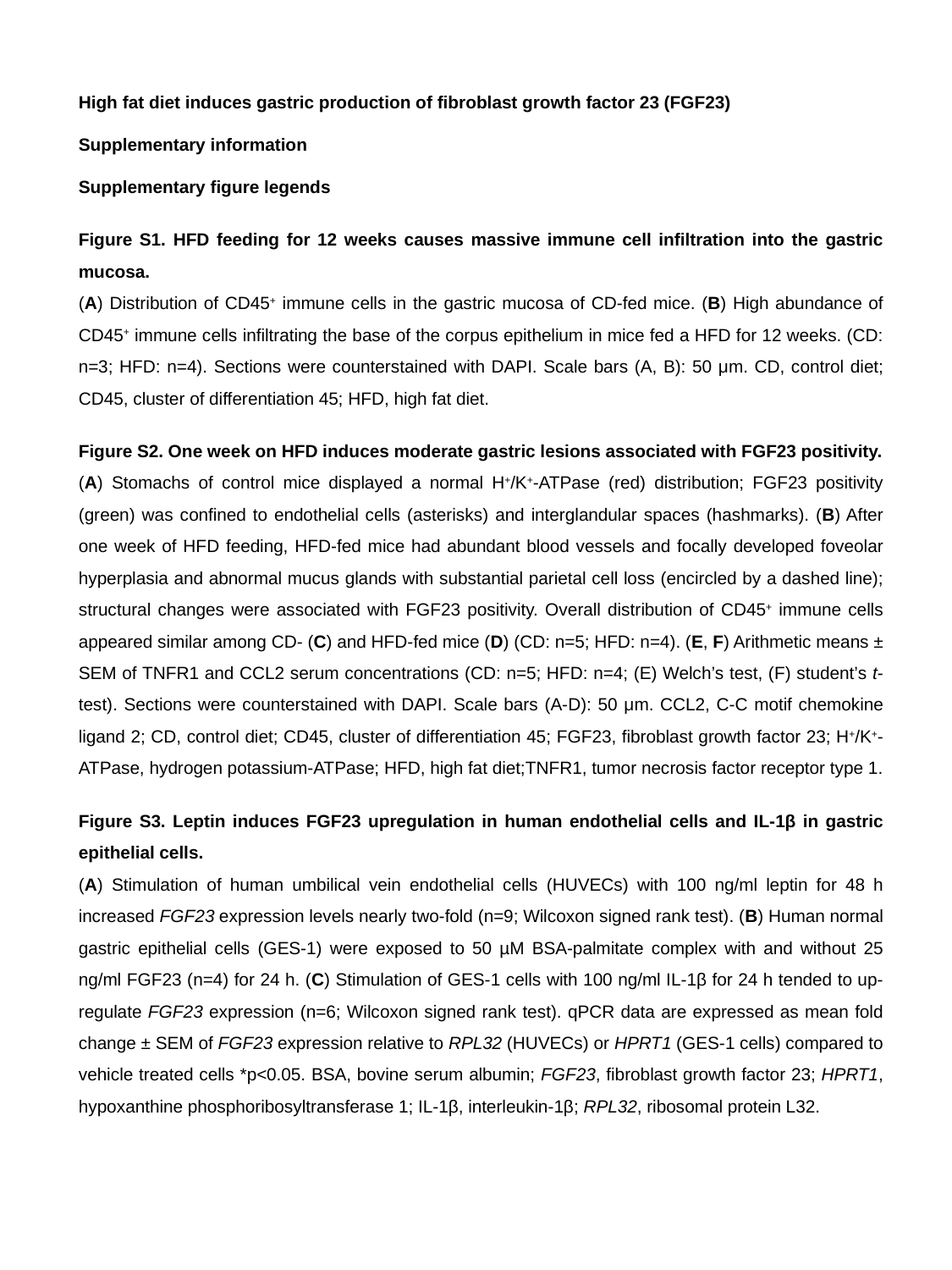

High fat diet induces gastric production of fibroblast growth factor 23 (FGF23)
Supplementary information
Supplementary figure legends
Figure S1. HFD feeding for 12 weeks causes massive immune cell infiltration into the gastric mucosa.
(A) Distribution of CD45+ immune cells in the gastric mucosa of CD-fed mice. (B) High abundance of CD45+ immune cells infiltrating the base of the corpus epithelium in mice fed a HFD for 12 weeks. (CD: n=3; HFD: n=4). Sections were counterstained with DAPI. Scale bars (A, B): 50 μm. CD, control diet; CD45, cluster of differentiation 45; HFD, high fat diet.
Figure S2. One week on HFD induces moderate gastric lesions associated with FGF23 positivity.
(A) Stomachs of control mice displayed a normal H+/K+-ATPase (red) distribution; FGF23 positivity (green) was confined to endothelial cells (asterisks) and interglandular spaces (hashmarks). (B) After one week of HFD feeding, HFD-fed mice had abundant blood vessels and focally developed foveolar hyperplasia and abnormal mucus glands with substantial parietal cell loss (encircled by a dashed line); structural changes were associated with FGF23 positivity. Overall distribution of CD45+ immune cells appeared similar among CD- (C) and HFD-fed mice (D) (CD: n=5; HFD: n=4). (E, F) Arithmetic means ± SEM of TNFR1 and CCL2 serum concentrations (CD: n=5; HFD: n=4; (E) Welch’s test, (F) student’s t-test). Sections were counterstained with DAPI. Scale bars (A-D): 50 μm. CCL2, C-C motif chemokine ligand 2; CD, control diet; CD45, cluster of differentiation 45; FGF23, fibroblast growth factor 23; H+/K+-ATPase, hydrogen potassium-ATPase; HFD, high fat diet;TNFR1, tumor necrosis factor receptor type 1.
Figure S3. Leptin induces FGF23 upregulation in human endothelial cells and IL-1β in gastric epithelial cells.
(A) Stimulation of human umbilical vein endothelial cells (HUVECs) with 100 ng/ml leptin for 48 h increased FGF23 expression levels nearly two-fold (n=9; Wilcoxon signed rank test). (B) Human normal gastric epithelial cells (GES-1) were exposed to 50 µM BSA-palmitate complex with and without 25 ng/ml FGF23 (n=4) for 24 h. (C) Stimulation of GES-1 cells with 100 ng/ml IL-1β for 24 h tended to up-regulate FGF23 expression (n=6; Wilcoxon signed rank test). qPCR data are expressed as mean fold change ± SEM of FGF23 expression relative to RPL32 (HUVECs) or HPRT1 (GES-1 cells) compared to vehicle treated cells *p<0.05. BSA, bovine serum albumin; FGF23, fibroblast growth factor 23; HPRT1, hypoxanthine phosphoribosyltransferase 1; IL-1β, interleukin-1β; RPL32, ribosomal protein L32.

## Slide 2
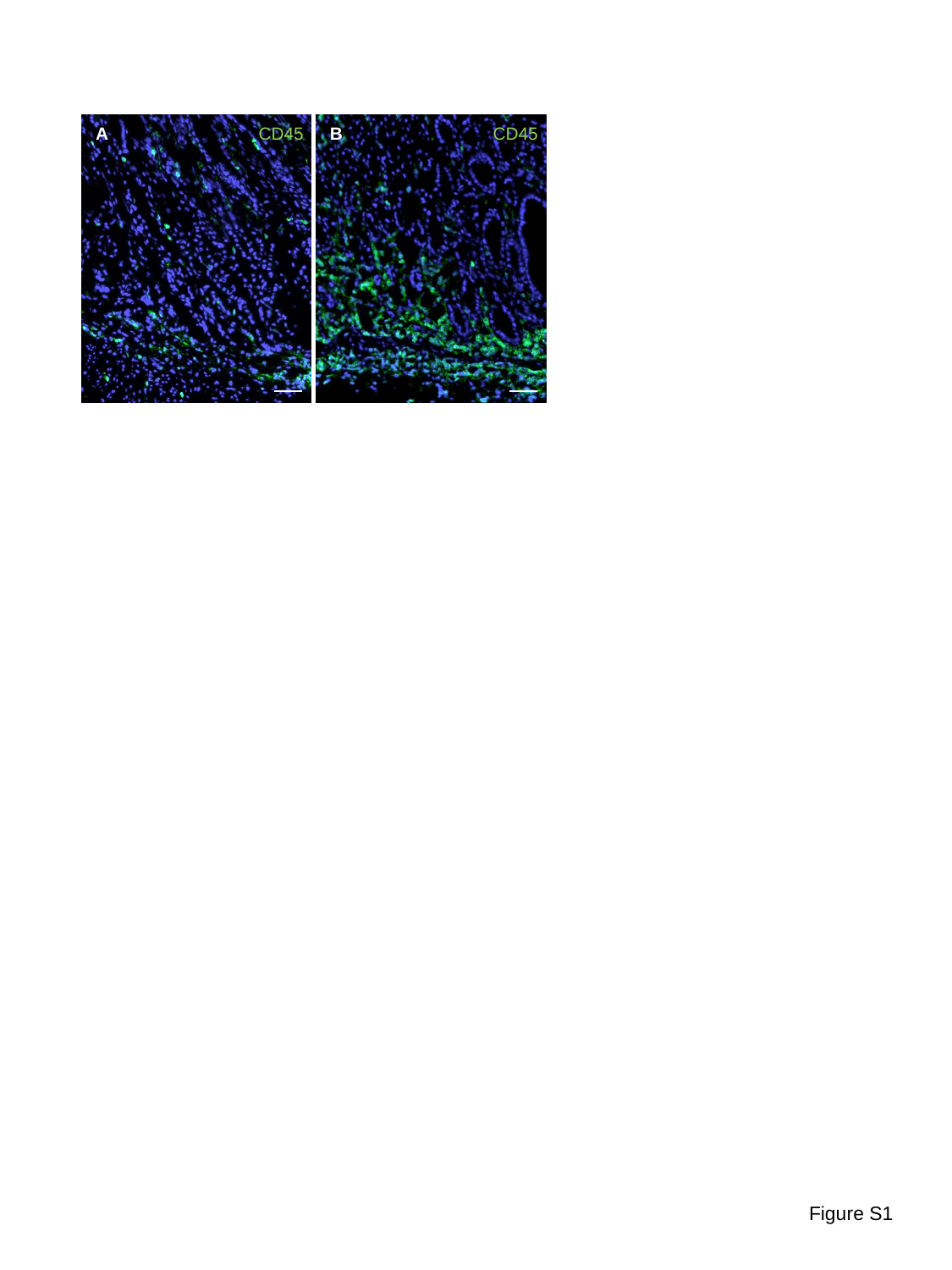

A
CD45
CD45
B
Figure S1

## Slide 3
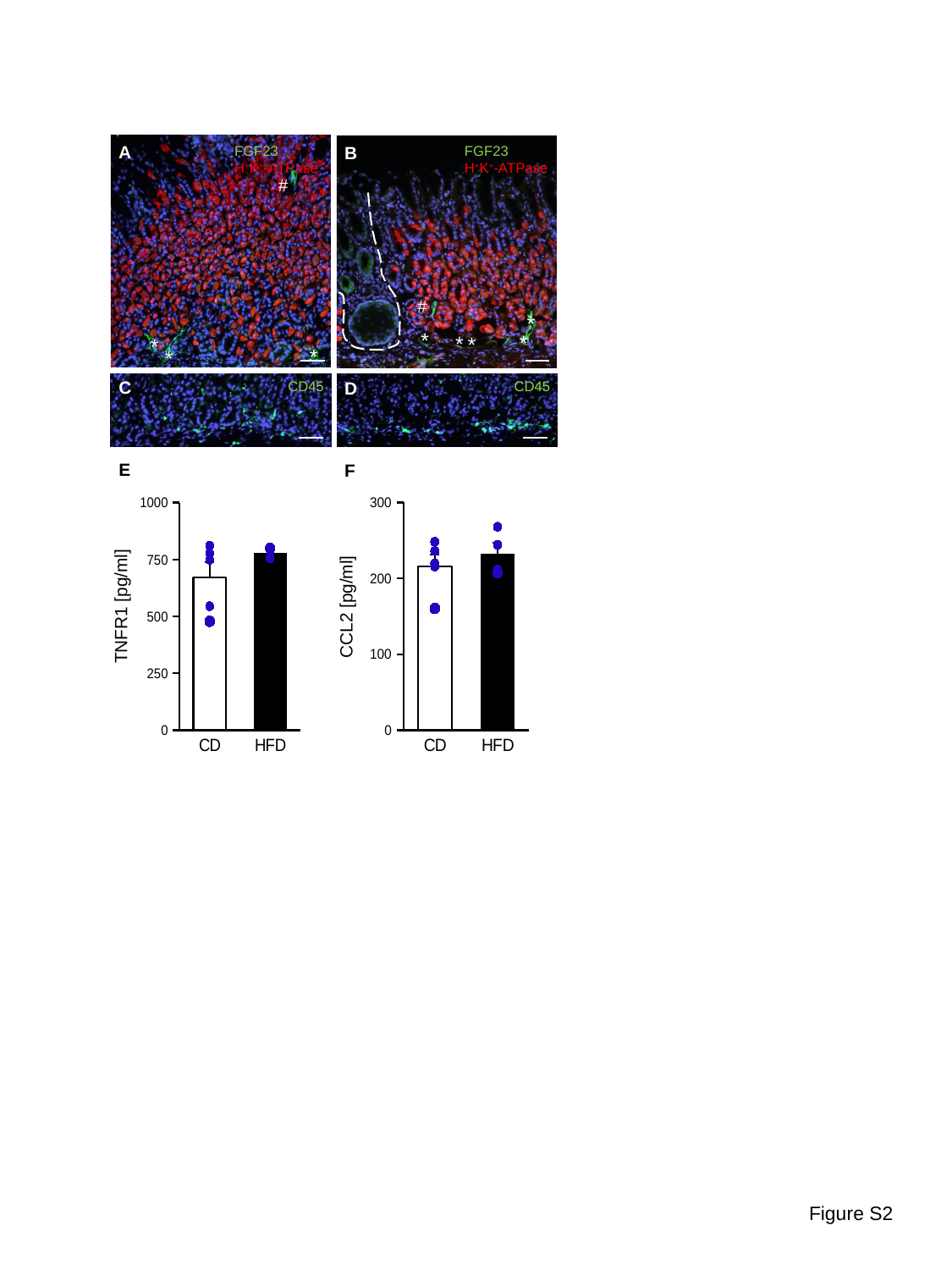

A
FGF23
H+K+-ATPase
B
#
#
*
*
*
*
*
*
*
*
C
D
CD45
CD45
### Chart
| Category | | | | | | | |
|---|---|---|---|---|---|---|---|
| CD | 215.94093890028444 | 215.582124732316 | 235.992109181346 | 160.375589140898 | 248.059057532266 | 219.695813914596 | None |
| HFD | 232.6772239923915 | 244.050798172878 | 267.902316294894 | 207.30407557509 | 211.451705926704 | None | None |
### Chart
| Category | | | | | | | |
|---|---|---|---|---|---|---|---|
| CD | 671.4827122606732 | 544.748608320398 | 748.61966406421 | 478.304839590882 | 809.874005804378 | 775.866443523498 | None |
| HFD | 778.928438294053 | 755.597878870028 | 803.435546373614 | 798.52238182594 | 758.15794610663 | None | None |E
F
TNFR1 [pg/ml]
CCL2 [pg/ml]
FGF23
H+K+-ATPase
Figure S2

## Slide 4
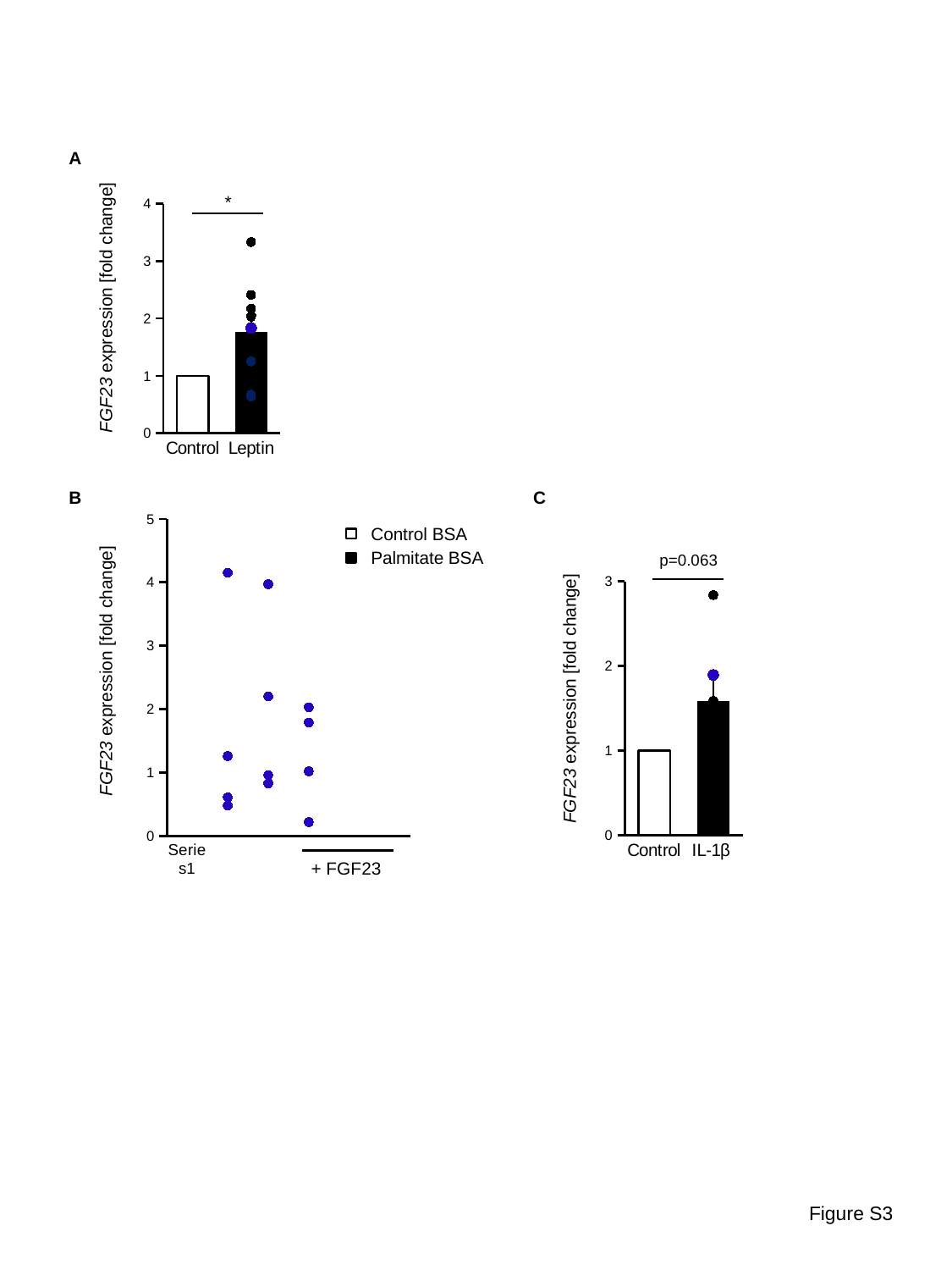

A
### Chart
| Category | | | | | | | | | | |
|---|---|---|---|---|---|---|---|---|---|---|
| Control | 1.0 | None | None | None | None | None | None | None | None | None |
| Leptin | 1.77 | 2.41 | 2.17 | 1.83 | 3.33 | 1.6 | 2.03 | 1.25 | 0.64 | 0.67 |*
FGF23 expression [fold change]
[unsupported chart]
B
C
Control BSA
### Chart
| Category | | | | | | | |
|---|---|---|---|---|---|---|---|
| Control | 1.0 | None | None | None | None | None | None |
| IL-1β | 1.5866666666666667 | 2.838 | 1.176 | 1.893 | 1.183 | 0.844 | 1.586 |
Palmitate BSA
p=0.063
FGF23 expression [fold change]
FGF23 expression [fold change]
+ FGF23
Figure S3
